# A Case of Acute Colonic Pseudo-Obstruction and Anastomotic Leakage After Sigmoidectomy for Sigmoid Volvulus

**DOI:** 10.7759/cureus.61133

**Published:** 2024-05-26

**Authors:** Kazumasa Nakamura, Shunsuke Sakuraba, Kohei Koido, Hiroyuki Hazama, Kou Ohata

**Affiliations:** 1 Gastrointestinal Surgery, Shizuoka General Hospital, Shizuoka, JPN

**Keywords:** sigmoid volvulus, postoperative ileus, suture failure, colonic obstruction, acute colonic pseudo-obstruction

## Abstract

Acute colonic pseudo-obstruction (ACPO) is characterized by significant colonic distension without a mechanical obstruction. We present a case of an 83-year-old male who developed ACPO following laparoscopic surgery for sigmoid volvulus. This report details the patient's postoperative journey, highlighting the diagnostic and management challenges encountered. Despite various medical interventions, the patient's condition necessitated further surgical attention due to complications. This case underscores the importance of early diagnosis and aggressive management in ACPO to prevent life-threatening consequences and improve patient outcomes.

## Introduction

Acute colonic pseudo-obstruction (ACPO) is a form of colonic obstruction caused by functional failure, and it exists in the absence of a physical origin of obstruction in the intestinal tract [1]. ACPO is often secondary to underlying diseases such as surgery, infection, toxic diseases, drugs, cardiac diseases, trauma, and cerebrovascular disorders [1,2]. Most of the surgical cases have been reported after cesarean sections, pelvic surgery, or cardiac surgery [1,2], and a few cases have occurred after laparoscopic surgery [3-5]. In this report, we present a case of an 83-year-old male who developed ACPO following laparoscopic surgery for recurrent sigmoid volvulus. The aim is to highlight the importance of early diagnosis and aggressive management in improving the prognosis of ACPO. Initial conservative treatments, including colonoscopic decompression with transanal tube placement, were unsuccessful. The patient ultimately required emergency surgery due to intestinal perforation. This case underscores the diagnostic and management challenges and the critical need for prompt and effective intervention in ACPO cases.

## Case presentation

An 83-year-old male presented with a chief complaint of abdominal distention. He had a history of hypertension and a cerebral infarction. In September 2022, while hospitalized for a left internal cerebral infarction, he experienced abdominal distention and was diagnosed with sigmoid colon volvulus via abdominal computed tomography (CT) imaging. On the same day, endoscopic derotation was performed. His symptoms improved temporarily; however, in November 2022, he was diagnosed with recurrent sigmoid colon volvulus due to recurrent abdominal distention. Another endoscopic derotation was attempted with difficulty. The patient was admitted to our hospital with a colorectal tube placed for decompression and was then referred to our department for surgery due to the difficulty in performing endoscopic procedures for the recurrent sigmoid volvulus.

**Figure 1:**
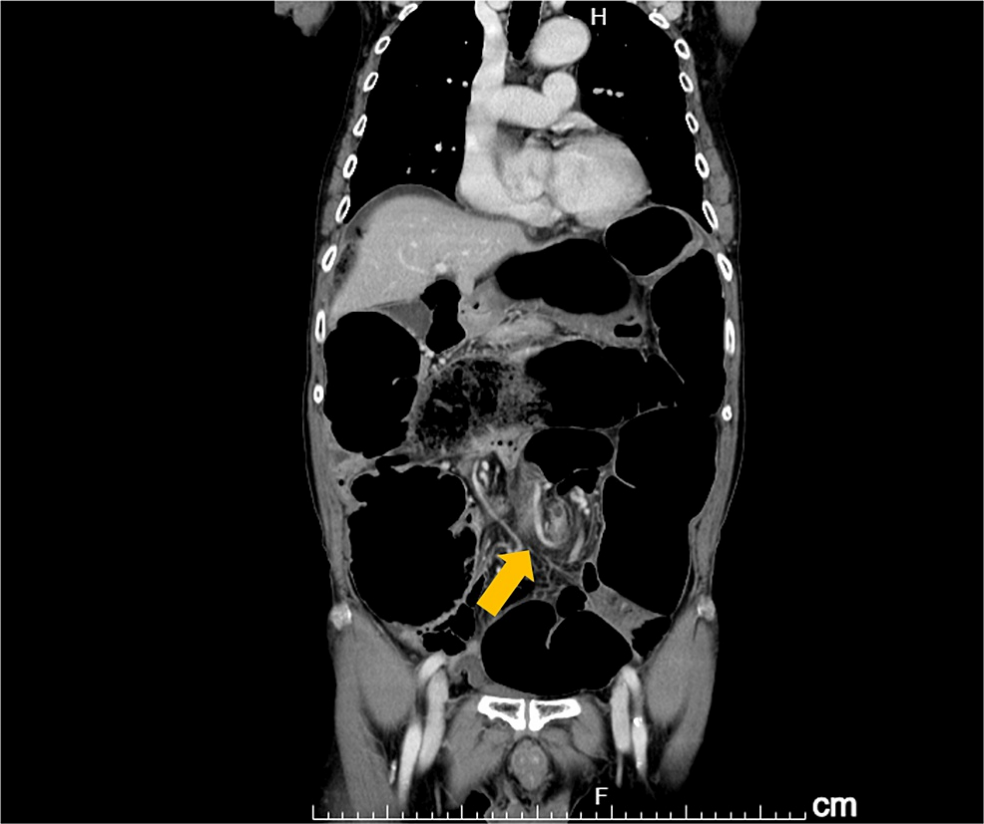
CT imaging indicating sigmoid colon axis torsion at the arrowed location

**Figure 2:**
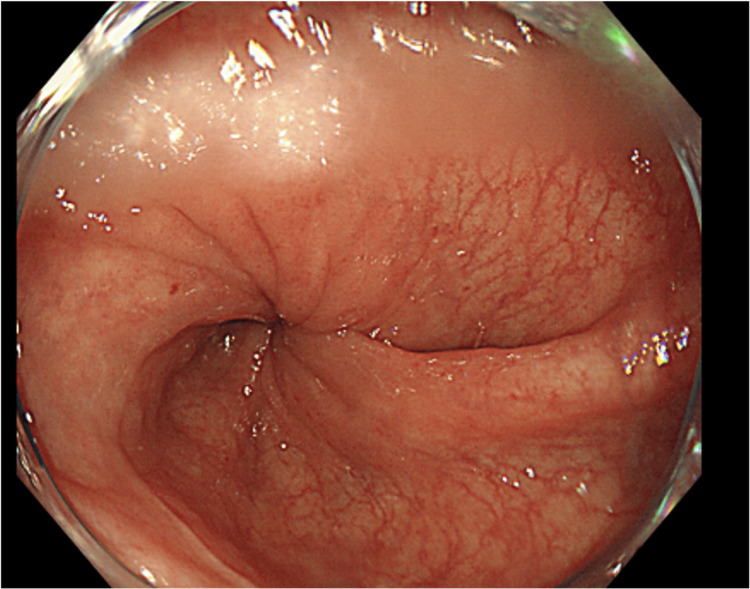
Colonoscopy revealing axial torsion of the sigmoid colon

His status at admission was as follows: height 148 cm, weight 42 kg, and body mass index 19.1. A marked distention of his abdomen was evident; however, no tenderness was noted. He was orally administered clopidogrel, Daikenchuto, a traditional Japanese herbal medicine, magnesium oxide, and azelnidipine. An abdominal X-ray revealed dilation of the colon. A contrast-enhanced CT of the abdomen indicated sigmoid volvulus (Figure 1). There were no findings on CT suggestive of ischemia or perforation of the intestinal tract. A colonoscopy confirmed a sigmoid volvulus (axial torsion condition) (Figure 2). A laparoscopic sigmoidectomy was chosen due to the ineffectiveness of conservative treatments.

Initial surgical findings

The patient underwent a laparoscopic sigmoidectomy to treat the sigmoid volvulus. Intraoperative findings confirmed that the sigmoid colon could be sufficiently elevated laparoscopically. Subsequently, a small incision was made, and a sigmoidectomy was performed. A functional end-to-end anastomosis was performed. The surgery took 79 minutes, and a small amount of bleeding was noted.

Postoperative course

On postoperative day 3, postoperative abdominal distension was diagnosed as due to postoperative paralytic ileus, and decompression using a nasogastric tube was performed. The patient was treated with laxatives; however, his symptoms did not improve. He was suspected of having ACPO on postoperative day 7, which was decompressed via colonoscopy with the placement of a transanal tube. The patient's symptoms improved, and diarrhea was observed. The patient was initially scheduled for discharge around postoperative day 14. However, during hospitalization, he contracted COVID-19, which required extended isolation and additional treatment. As a result, his hospital stay was prolonged. Eventually, the patient was discharged on postoperative day 26 with no complaints.

**Figure 3:**
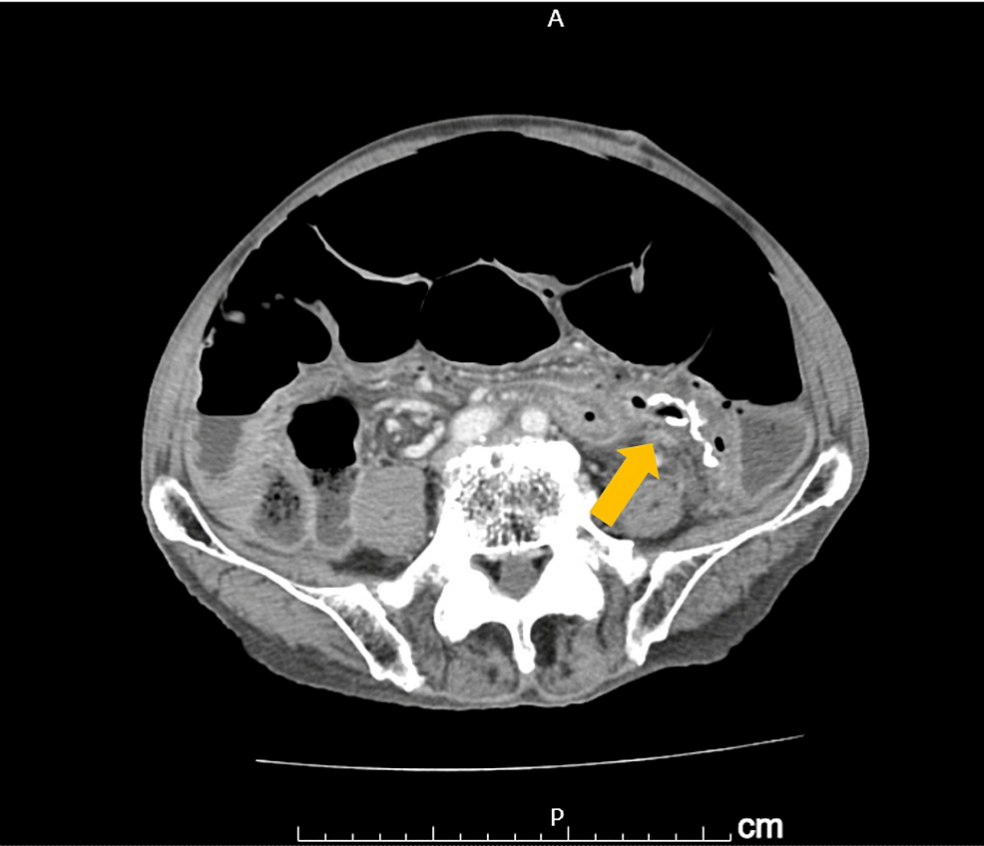
CT scan showing free air and ascites around the anastomotic site

However, he was readmitted on postoperative day 37 with abdominal distension and melena. A CT scan revealed extensive dilatation of the entire rectum and colon, suggesting a relapse of ACPO. After endoscopic decompression and placement of a transanal tube, the abdominal distention improved, and the patient was able to defecate. Despite these improvements, the patient experienced repeated symptom flare-ups and required ongoing medical attention. Therefore, he remained hospitalized for further observation and conservative treatment. On the 47th day after surgery, abdominal pain worsened rapidly. A CT scan showed intra-abdominal free gas and ascites, indicating suture failure at the sigmoid colon anastomosis site (Figure 3).

Observations during revision surgery

A laparotomy was performed through a midline abdominal incision. The entire abdominal cavity was contaminated with turbid ascites. A perforation was observed at the sigmoid colon anastomosis site. The leaked anastomosis site was resected, and a descending single-hole colostomy was created following Hartmann's procedure.

Postoperative course

The patient was discharged 18 days after the reoperation without any postoperative complications. Given the patient's overall health condition and his preference, the decision was made not to close the ostomy, opting for a permanent stoma. One year and six months have passed since the operation, with no worsening of abdominal symptoms.

## Discussion

ACPO, also known as Ogilvie's syndrome, is characterized by acute pseudo-obstruction of the colon without mechanical stenosis, first described by Ogilvie in 1948 [1]. It predominantly affects males aged 60 years or older and is often precipitated by underlying factors such as surgery, infection, toxic diseases, drugs, cardiac disease, trauma, and cerebrovascular disease [1,2]. Although most cases have been reported following major surgeries, such as coronary artery bypass, organ transplantation, cesarean delivery, and orthopedic procedures, few reports link ACPO to laparoscopic or robot-assisted surgery [3-5].

The exact etiology of ACPO remains unknown, but the prevailing theory is that it results from a disruption in the autonomic nervous system's control over colonic motility. The colon receives sympathetic innervation from the celiac and mesenteric plexuses and parasympathetic innervation from the vagus nerve (proximal colon) and pelvic nerve (distal colon) [6]. Disruption in these pathways can lead to motility imbalances, resulting in ACPO [1,2,7]. In the case presented, sigmoid volvulus occurred during the treatment for cerebral infarction, requiring endoscopic decompression. Post-surgical dilation persisted, likely due to prolonged dilation from repeated sigmoid volvulus or disruption of enteric innervation.

Clinically, ACPO manifests as significant abdominal distention due to colonic dilation. Symptoms can include abdominal pain (80% of cases), nausea and vomiting (60%), constipation (50%), and, paradoxically, diarrhea (40%) [1,8]. Hypokalemia, resulting from diarrhea-induced potassium loss, complicates treatment, as these cases often do not respond well to standard decompression techniques or treatments such as neostigmine [9].

The American Society for Gastrointestinal Endoscopy's guidelines for ACPO treatment recommend initial imaging to rule out mechanical obstruction, ischemia, or perforation [10]. If no improvement is observed within three days of conservative management, pharmacological intervention with neostigmine should be considered [10,11]. In the presented case, neostigmine was contraindicated due to the patient's cardiac history. When pharmacotherapy is not indicated, decompression can be performed via colonoscopy or a transanal tube. Both methods are considered safe [12,13]; therefore, colonoscopic decompression and transanal tube placement were used. Although there is no concrete evidence supporting transanal drainage, it was used in this case as it has been observed to improve symptoms and X-ray findings when applied. These interventions provided temporary relief but were insufficient for long-term management, highlighting the necessity of timely and appropriate escalation of care. In the presented case, transanal tube placement was employed twice, initially and after a relapse, to manage the colonic distension effectively.

Surgical intervention becomes imperative in the presence of colonic ischemia, perforation, or peritonitis, or when conservative and pharmacological treatments fail [10]. The risk of ischemia and perforation increases significantly when the colonic diameter exceeds 12 cm or when dilation persists beyond six days, with associated mortality rates ranging from 36% to 44% [1,14]. In this case, despite the colon diameter being less than 10 cm, the persistent dilation over approximately 40 days led to complications, including anastomotic leakage and generalized peritonitis, necessitating emergency surgery.

It is essential to distinguish between ACPO and anastomotic leakage. In this case, the initial diagnosis of ACPO was based on the absence of clinical signs of anastomotic failure, such as localized peritonitis or sepsis, and the presence of colonic distension without evidence of mechanical obstruction. The improvement of symptoms following colonoscopic decompression and the recurrence of distension further supported the diagnosis of ACPO rather than an anastomotic leak. The subsequent development of anastomotic leakage occurred later in the clinical course, following the persistent colonic distension, which is a known complication in cases of untreated or recurrent ACPO.

Surgical options vary depending on the severity and extent of the condition, from creating a stoma to performing a total or subtotal colectomy [14]. Here, a subtotal resection was avoided despite extensive colonic dilation, focusing instead on resection with a leaked anastomosis site and creating a single-hole stoma. This approach successfully alleviated symptoms without necessitating a more extensive resection. Given the patient's overall health status and preference, the decision was made to maintain the stoma permanently. The patient remained stable, with no recurrence of colonic dilation observed one year and six months post-surgery.

This case underscores the importance of early and aggressive management of ACPO to prevent severe complications and improve patient outcomes. It also highlights the challenges of managing postoperative complications and the need for a tailored approach to each patient's unique circumstances.

## Conclusions

This case demonstrates the complexity of managing ACPO following laparoscopic surgery for recurrent sigmoid volvulus. Despite initial conservative treatments providing temporary relief, the patient's condition deteriorated, necessitating further surgical intervention due to complications, including anastomotic leakage. Early diagnosis and aggressive management were crucial in preventing life-threatening consequences and improving patient outcomes. The patient remained stable with no further worsening symptoms over a year and six months post-operation. This case highlights the importance of timely surgical intervention in cases of recurrent ACPO and the challenges of managing postoperative complications.

## References

[1] Vanek VW, Al-Salti M (1986). Acute pseudo-obstruction of the colon (Ogilvie's syndrome). An analysis of 400 cases. Dis Colon Rectum.

[2] Wells CI, O'Grady G, Bissett IP (2017). Acute colonic pseudo-obstruction: a systematic review of aetiology and mechanisms. World J Gastroenterol.

[3] Cebola M, Eddy E, Davis S, Chin-Lenn L (2015). Acute colonic pseudo-obstruction (Ogilvie's syndrome) following total laparoscopic hysterectomy. J Minim Invasive Gynecol.

[4] Orfanelli T, Chung S, Kohut A, Gibbon D, Leiser A (2018). Ogilvie's syndrome after robotic-assisted radical hysterectomy for cervical cancer. J Minim Invasive Gynecol.

[5] Inagaki Y, Matsuo K, Nakano Y, Kondo T (2021). Acute colonic pseudo-obstruction and rapid septic progression after transabdominal preperitoneal hernia repair: a case report. BMC Surg.

[6] Ogilvie H (1948). Large-intestine colic due to sympathetic deprivation; a new clinical syndrome. Br Med J.

[7] Saunders MD (2007). Acute colonic pseudo-obstruction. Best Pract Res Clin Gastroenterol.

[8] Jetmore AB, Timmcke AE, Gathright JB Jr, Hicks TC, Ray JE, Baker JW (1992). Ogilvie's syndrome: colonoscopic decompression and analysis of predisposing factors. Dis Colon Rectum.

[9] Bazerbachi F, Haffar S, Szarka LA, Wang Z, Prokop LJ, Murad MH, Camilleri M (2017). Secretory diarrhea and hypokalemia associated with colonic pseudo-obstruction: a case study and systematic analysis of the literature. Neurogastroenterol Motil.

[10] Naveed M, Jamil LH, Fujii-Lau LL (2020). American Society for Gastrointestinal Endoscopy guideline on the role of endoscopy in the management of acute colonic pseudo-obstruction and colonic volvulus. Gastrointest Endosc.

[11] Valle RG, Godoy FL (2014). Neostigmine for acute colonic pseudo-obstruction: a meta-analysis. Ann Med Surg (Lond).

[12] Haj M, Haj M, Rockey DC (2018). Ogilvie's syndrome: management and outcomes. Medicine (Baltimore).

[13] Book T, Kirstein MM, Schneider A, Manns MP, Voigtländer T (2020). Endoscopic decompression of acute intestinal distension is associated with reduced mortality in critically ill patients. BMC Gastroenterol.

[14] Alavi K, Poylin V, Davids JS (2021). The American Society of Colon and Rectal Surgeons Clinical Practice Guidelines for the Management of Colonic Volvulus and Acute Colonic Pseudo-Obstruction. Dis Colon Rectum.

